# Chronic periodontitis and its possible association with oral squamous cell carcinoma – a retrospective case control study

**DOI:** 10.1186/1746-160X-9-39

**Published:** 2013-12-09

**Authors:** Maximilian Moergel, Peer Kämmerer, Adrian Kasaj, Evangelia Armouti, Abdulmonem Alshihri, Veronika Weyer, Bilal Al-Nawas

**Affiliations:** 1 Department of Oral and Maxillofacial Surgery, University Medical Centre Mainz, Augustusplatz 2, Mainz 55131, Germany; 2 Harvard Medical School, Boston, USA; 3 Department of Operative Dentistry, University Medical Centre Mainz, Mainz, Germany; 4 Department of Restorative and Biomaterial Sciences, Harvard School of Dental Medicine, Boston, USA; 5 Institute of Medical Biostatistics, Epidemiology and Informatics (IMBEI), University Medical Centre Mainz, Mainz, Germany

**Keywords:** Chronic periodontitis, Bone loss, Oral squamous cell carcinoma, Retrospective, Primary prevention

## Abstract

**Introduction:**

Different inflammatory processes may trigger the development of malignancies. Therefore, the aim of the present study was to evaluate a potential association between radiological determined chronic periodontitis (CPA) and oral squamous cell carcinoma (OSCC).

**Methods:**

In a retrospective study, OSCC-patients and a control group without malignant tumors were radiographically examined for bone loss. Via telephone survey and questionnaire, general clinical data on the individual oral hygiene and concomitant diseases together with tobacco and alcohol use were assessed and data were compared between the groups.

**Results:**

178 OSCC-patients and 123 controls were included. In univariate analysis, a statistically relevant higher mean bone loss was seen in the OSCC group (4.3 mm (SD: 1.8; 95% confidence interval (CI): 4-4.6) vs. 2.9 mm (SD: 0.7; 95% CI: 2.8-3); p < 0.001)). This was confirmed in a multivariate regression model (OR: 2.4, 95% CI: 1.5-3.8; p < 0.001). A history of periodontal treatment was associated with significantly reduced OSCC risk (p < 0.001; OR: 0.2, CI: 0.1-0.5).

**Conclusions:**

CPA is a common disease and the monitoring as well as the treatment of such a chronic oral inflammation may be beneficial in reducing one potential cause of OSCC. Therefore, further clinical studies on oral neoplasms should consider clinical periodontal parameters as well.

## Introduction

Oral squamous cell carcinoma (OSCC) accounts for about 90% of all oral malignancies causing a significant number of mutilations and cancer related deaths each year. As the OSCC related survival rate has not significantly changed during the last decades and remains nearly 50% and is even worse for different subtypes and more advanced forms [1], assessment of future strategies for primary prevention is of high clinical relevance. This could lead to the recognition of the pre-malignant oral lesions and neoplastic transformation at initial stages with a positive impact on the outcome [2]. Well-known risk factors for the development of OSCC are tobacco, alcohol, betel quid ingestion, malnutrition as well as viral infections [1,3]. For chronic inflammation, an increased risk for malignant transformation of the affected epithelium is assumed [4]. For example, oral lichen planus, a common inflammatory disease of autoimmune etiology, has a frequency of malignant transformation of 0.4-5.3% [5]. Likewise, Barrett’s esophagus, a chronic inflammation of the upper gastrointestinal tract, is a precancerous condition for the development of adenocarcinomas [6]. Bacterial and viral infections may also induce a chronic inflammation with the potential for malignant transformation. Helicobacter pylorus is a bacterium that is correlated with acute and chronic gastritis and with malignant gastric neoplasms [7,8]. There is evidence for an association between infections with Salmonella typhi and cancer of the hepatic system [9]. Other infections with bacteria such as Citrobacter rodentium in mice [10], Chlamydia pneumonia [11] and Streptococcus bovis [12] are likewise positively correlated with the incidence of malignancies. For these events, carcinogenesis represents the end point of a cascade in which bacterial toxin, inflammatory factors and mediators cause direct or indirect DNA damage that consecutively leads to cell transformation [7,13].

Chronic periodontitis is a multi-factorial, opportunistic inflammation of the periodontium mostly caused by gram-negative, anaerobic bacteria. Microbial toxins, proteases and endotoxins are secreted, inducing an inflammation through stimulation of monocytes with further excretion of mediators like prostaglandin E2, thromboxane B2, interleukin-1, -6, -8, -17, tumor necrosis factor and collagenases [14,15]. Fibroblasts and osteoclastic cells are stimulated and matrix metallo proteinases disintegrate collagen fibers and promote bone resorption [16]. In general, the disease shows a slow progression with limited pathology at early stages. The clinical manifestations are increased gingival pocket depth with loss of gingival attachment and destruction of the underlying alveolar bone. Further symptoms may include abscess formation and increased teeth mobility with subsequent teeth loss. A radiographic assessment in order to diagnose chronic periodontitis is possible and was used in similar studies [17,18].

An induction of OSCC by such chronic bacterial inflammation appears possible since the involved inflammatory mediators, cytokines and bacterial toxins have shown to have a potential for malignant transformation in vitro [13,15,19]. For tongue cancer, a positive correlation was already shown in a retrospective clinical setting [17]. Since chronic periodontitis is a very common disease and anti-inflammatory therapies have previously shown a positive impact on the evolution of several malignant diseases [20,21], a clinical relevance of a putative connection between chronic periodontitis and malignant transformation would be of great interest. The primary hypothesis of this study was that there is an association between chronic periodontitis and oral squamous cell carcinoma. Therefore, this correlation between radiological signs of chronic periodontitis and prevalence of OSCC was examined. As objective clinical parameters, radiographic bone loss in patients with and without OSCC prior periodontal treatment were determined and compared. As secondary criteria, mouth hygiene, tobacco and alcohol ingestion as well as concomitant diseases were assessed.

## Materials and methods

### Patients

After a positive appraisal of the local ethical committee of Rhineland-Palate (No. 837.233.08 (6230)), a group of patients with OSCC and a control group, each treated in our department between 01/2002 and 12/2010, were examined retrospectively in the time between 01/2011 and 01/2012. The inclusion criteria for the cancer group were as follows: untreated, primary OSCC limited to the area of periodontitis (proximal to gingiva and mandibular/maxillary alveolar mucosa) with preoperative panoramic x-ray; at least 6 remaining teeth; no other malignant tumors beside the oral cavity; no inflammatory diseases of the jaws as well as autoimmune disorders and/or infectious diseases; no prior intake of medication affecting the periodontium; no pregnancy; no trauma and/or fractures affecting the periodontium prior evaluation and informed consent. Patients in the control group had to be without malignant diseases in and beyond the oral cavity. All other criteria were the same as in the OSCC-group.

### Radiological evaluation

The radiological signs of chronic periodontitis were evaluated in a blinded manner by one trained observer (E. A.^a^). In brief, the mesial and distal bone loss of all non-retained teeth was determined using the panoramic x-ray with the software SIDEXIS XG (Sirona Dental Systems, Bensheim, Germany). For assessment of the bone loss, two vertical lines parallel to the axis of the tooth at the cementoenamel junction to the crestal bone were drawn at the mesial and distal aspect of each tooth, respectively. Horizontal lines orthogonal to the prior characterized vertical lines were drawn at the mesial and distal cementoenamel junction. The distance between the cross point of each vertical and its horizontal line to the crestal bone specified the respective bone loss. If a tooth had fillings and the cementoenamel junction was not visible, the lower edge of the filling served as reference point. An alveolar bone loss of less than 2 mm is generally considered as healthy periodontium [22]. Mean values (mm) were used for statistics. A second examiner (M. M.) reviewed the calculations in order to assure the reproducibility.

### Questionnaire

All patients received a questionnaire in order to obtain data with regards to age, gender, body weight and height together with the marital status and level of education. Furthermore, comprehensive assessment was documented including the habitual oral hygiene (frequency of teeth brushing, use of oral rinse and dental floss), date of the last dental examination, gingival bleeding, halitosis, periodontal treatment in the past as well as any existing dental prosthesis. Additionally, tobacco (packs/years) and alcohol use and any concomitant diseases were investigated. Moreover, the subjective stress profile prior to OSCC diagnosis and/or control examination was assessed by visual analogue scale (from 1 (none) to 10 (very)).

### Statistics

For descriptive analysis of categorial data, absolute and relative frequencies were calculated. For continuous data, minimum, maximum, median and mean values, skewness, quartiles and standard deviations were evaluated. Categorial data were visualized via bar charts, consistent data via boxplots. In the further explorative data analysis, Kolmogorov-Smirnov test was employed to test for a difference in the two groups. In cases of p-values <0.05, Mann-Whitney-U test and in cases of p-values >0.05, Students’ t-test for independent samples was employed. The influence of categorical variables was shown with chi-square tests and cross tables.

For confirmatory evaluation, a multivariable analysis was performed. For further differentiation of obtained data, a multiple logistic regression analysis was conducted. A global significance level was chosen to 0.05. Due to multiple testing the Bonferroni correction was applied. Four hypotheses were analyzed confirmatory, so the local significance level was 0.0125. All analyses were carried out with SPSS Statistics version 19 (IBM, Armonk, NY, USA).

## Results

### Patients

A total of 301 patients were included. The OSCC group consisted of 178 patients (female: n = 56, male: n = 122; mean age: 60 years (SD: ± 10.7; min: 39, max: 88)). Table 1 gives the locations of the respective OSCCs. For the control group, 123 patients (female: n = 65, male: n = 58; mean age: 57 years (SD: ± 9.7; min: 37, max: 80)) were evaluated. The ages of both groups were matched. Compared to the control group, the OSCC group consisted of significant more men (p < 0.001). The gender ratio within the OSCC group was 2.1 in the favor of men, whereas the gender ratio within the control group was nearly balanced (women: men = 1.12:1).

**Table 1:** Location of OSCCs

**Location**	
Mouth floor next to alveolar bone	n = 44 (25%)
Tongue next to alveolar bone	n = 41 (23%)
Alveolar bone mandible	n = 36 (20%)
Alveolar bone maxilla	n = 9 (5%)
Palate next to maxillary alveolar bone	n = 7 (4%)
Cheek next to alveolar bone	n = 6 (3%)
Multiple locations including proximity of alveolar bone	n = 35 (20%)

### Univariate analysis

#### Radiological evaluation

**Figure 1:**
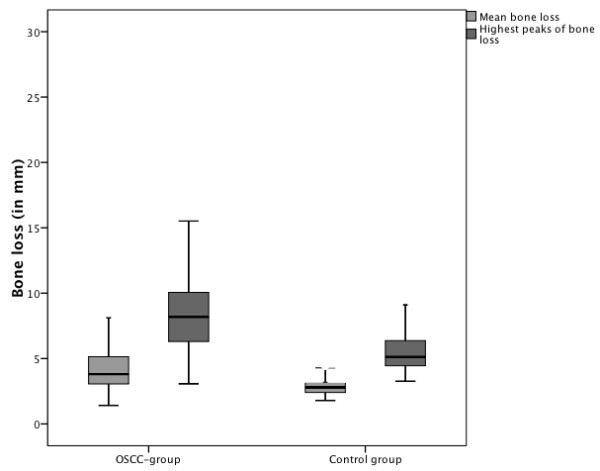
**Box plots showing the values for mean bone losses as well as the highest peaks of bone losses per patient in the OSCC and the control group.** Both differences were statistical significant.

In the OSCC group, the mean bone loss was 4.3 mm (SD: 1.8; 95% confidence interval (CI): 4-4.6) and 2.9 mm (SD: 0.7; 95% CI: 2.8-3) for the controls. This difference was statistically relevant (p < 0.001; Figure 1). The highest peaks of bone loss were seen in OSCC patients as well (OSCC mean 8.6 mm (SD: 3.3; 95% CI: 8.1-9.1) vs. controls with a mean of 5.5 mm (SD: 1.6; 95% CI: 5.2-5.8; p < 0.001; Figure 1)). In 46 OSCC patients (26%), the most distinct bone loss was observed in direct proximity to the malignant tumor. Whereas the highest total bone losses were seen from the postcanine region (67%) up to the second molar in all quadrants, the highest frequency of bone loss were in the precanine (17%) followed by the canine (10%) and the postmolar region (6%). This tendency was observed in both groups.

### Questionnaire

If the patients were not available, a telephone survey was carried out. Out of the 178 OSCC patients, 71 (40%) denied any oncological recall, 27 (15%) were dead to the time of evaluation and 11 (6%) rejected to fill out the questionnaire. Altogether, a positive response was obtained in 69 OSCC patients (39%) and in 129 patients (100%) of the control group. The BMI was comparable between the both groups (p = 0.719) and their marital status was similar (p = 0.05). OSCC patients had a statistically relevant decreased level of education compared to the controls (<12 years vs. >12 years; p = 0.021). Further information about dental hygiene, smoking and alcohol habits as well as concomitant diseases is given in Table 2. In brief, a statistically relevant higher amount of patients with frequent teeth brushing (p = 0.045) and dental floss use (p < 0.001) were seen in the control group. On contrary, OSCC patients used more products for oral rinse (p = 0.015). Compared to the controls, OC patients had statistically relevant less periodontal treatments (p = 0.002) and less dental rehabilitation (including crowns, fixed partial dentures, dental implants; p < 0,001). OSCC patients smoked statistically relevant more (p < 0.001) whereas in the control group more alcohol was consumed (p = 0.03 Table 2).

**Table 2:** Patient characteristics obtained from the questionnaire

	**OSCC**	**Control**	**p-value**
*Teeth brushing >1/d*	58/69 (84%)	115/123 (94%)	***0.045***
*Frequency of oral rinse/week*	6 (SD 8)	4 (SD 5)	***0.015***
*Use of dental floss*	21/69 (30%)	76/123 (62%)	***<0.0001***
*Time to last dental examination ≤ 6 months*	50/69 (72%)	93/123 (76%)	0.588
*Gum bleeding*	5/69 (7%)	3/123 (2%)	0.138
*Halitosis*	2/69 (3%)	2/123 (2%)	0.443
*Periodontal treatment*	18/69 (26%)	60/123 (49%)	***0.002***
*Dental prosthesis*	54/69 (78%)	119/123 (97%)	***<0.0001***
*Tobacco/pack years*	30.7 (SD 34)	9.3 (SD 17.7)	***<0.0001***
*Alcohol*	38/69 (55%)	87/123 (71%)	**0.03**
*Concomitant diseases*			
*Coronary heart diseases*	19/69 (28%)	35/123 (29%)	1
*Diabetes mellitus*	4/69 (6%)	3/123 (2.4%)	0.253
*Pulmonary diseases*	4/69 (6%)	4/123 (3.3%)	0.461
*Apoplexy in anamnesis*	3/69 (4%)	5/123 (4%)	1
*Autoimmune disorders*	2/69 (3%)	6/123 (5%)	0.453
*Osteoporosis*	1/69 (1%)	3/123 (2,4%)	1
*Gastric diseases*	2/69 (3%)	1/123 (0.8%)	0.294
*Stress before examination (VAS)*	4.6 (SD 2.5)	4.8 (SD 2.8)	0.619

Significant differences are given in bold letters. For the parameter “frequency of oral rinse/week”, “tobacco/pack years” and “stress before examination (VAS)”, mean values with standard deviations (SD) are given.

### Multivariate analysis

In the multiple logistic regression model including gender, age, decayed, missing and filled teeth, bone loss as well as prior periodontal therapy, tobacco (packs/years) and alcohol abuse, the radiological parameter “mean bone loss” was still identified to be an independent risk factor for the presence of OSCC (p < 0.001; OR 2.4, 95% CI 1.5-3.8). In addition and with congruence to the clinical data it could be shown that prior periodontal treatment significantly reduced the risk of OSCC presence (p < 0.0001; OR 0.2, 95% CI 0.1-0.5). Smoking had a significant (p < 0.001) but clinical not relevant effect (CI 1-1.1) on the OSCC incidence (p < 0.001; OR 1, 95% CI 1-1.1; Table 3).

**Table 3:** Variables of the multiple logistic regression models together with each Odds Ratio (OR), 95% confidence interval and p-values

**Variable**	**OR (95% CI)**	**p-value**
*Gender*	0.7 (0.3-1.5)	0.33
*Age*	1 (1-1.1)	0.4
*Teeth with caries*	1.3 (0.8-2.2)	0.38
*Teeth with fillings*	0.9 (0.9-1)	0.09
*Missing teeth*	1 (0.9-1)	0.16
*Mean bone loss*	2.4 (1.5-3.8)	<0.001
*Periodontal treatment*	0.2 (0.1-0.5)	<0.001
*Alcohol*	1 (0.9-1)	0.28
*Tobacco/pack years*	1 (1-1.1)	<0.001

## Discussion

Primary prevention strategies and early diagnosis of OSCC are pivotal for the individual patient. Consequently, delayed diagnosis may result in advanced stages with increased morbidity and mortality. Thus, it would be of great clinical benefit if patients with a high-risk profile for the development of OSCC could be identified and integrated in clinical recall programs with short clinical follow-up frequency. Beside the generally accepted tobacco and alcohol model, the discussion about bacterial infection and chronic mucosal inflammations draws the process of inflammation itself more and more into focus as an independent risk factor for oral malignant transformation. Accordingly, the present retrospective study analyzed bone loss as an objective clinical parameter for chronic periodontitis as a potential risk factor for the presence of OSCC in a comparable high number of patients. Radiologically measured “mean” and “highest” bone loss was correlated to the presence of oral cancer in a case-control design. The radiographic assessment is an established method to evaluate periodontitis history and was used in similar studies [17,18]. Nevertheless, it is a major limitation of this study that only radiographic features were assessed. Additionally, as the respective x-rays of OSCC-patients were available before surgery only, no earlier diagnosis of chronic periodontitis at two different time points was possible. Further limitations are the low response rate of the questionnaire and the significant higher number of males in the test group. Therefore, further prospective clinical studies are needed.

In the present investigation “mean” and “highest” bone loss could be confirmed in univariate and multivariate analysis as independent risk factor for OSCC. As the alveolar bone resorption is a very slow progress in general [23], it can be hypothesized that there was a chronic periodontitis prior to cancer diagnosis. This underlines the high clinical relevance to treat oral chronic inflammations and is in accordance to the study of *Tezal et al.*[17]*.* In contrast to the latter study, the present patient population was larger, patients of both genders and cancer sites, other than the tongue, were included. Nevertheless, a possible bone loss due to spreading of the cancerous lesions has to be taken into account as well.

A general association between inflammatory processes and malignant tumors is known [24]. Such long lasting processes in oral as well as extraoral sites are supposed to induce tumors of different kind [5,6]. *Parkin* recently quoted that nearly 18% of all malignant diseases worldwide are possibly caused by inflammation [25]. Chronic periodontitis is defined as a chronic inflammation mainly caused by complex interacting gram-negative bacteria in the dental biofilm. The continuous release of toxins and inflammatory markers leads to a degradation of periodontal connective tissue. Genetic changes with subsequent malignant transformation in cases of chronic inflammations with gram negative bacteria were described previously [19]. For chronic periodontitis, an additional, synergistically viral involvement is likely [25] and deep periodontal pockets have been suggested to be a reservoir for HPV, cytomegalovirus and Ebstein-Barr virus [26,27]. In summary, induction of malignant tumors via viral or bacterial agents is known [7,28,29]. An association between chronic periodontitis and OSCC is explainable by direct toxic effects of microorganisms and their products as well as through activated inflammatory cells [7,18,30]. Additionally, modulation of the immune system and facilitation of tumor growth due to soft and hard tissue destruction by chronic periodontitis has to be considered.

In addition to the positive correlation between chronic periodontitis and OSCC, we obtained a hint that periodontal therapies including mechanical scaling and adjunctive antimicrobial therapies may have a protective impact on OSCC incidence. Of course, it has to be taken into consideration, that this assumption is based on a bad response rate of 39% of all patients with OSCC and we do not have information considering the length and the success of the respective therapy. The general benefit of anti-inflammatory therapies by the means of cancer protection in extra-oral sites was likewise proven previously [20,21,31]. Especially, the inhibition of cyclooxygenase-2 that may lead to a decreased loss of periodontal attachment seems to be hereby of importance [32]. The clinical impact is high, since mechanical removal of supra- and sub-gingival debris by scaling and root planning techniques is able to eliminate the cause of inflammation and with a periodontal recall program the disease may be stabilized. In the multivariate regression model, neither dental hygiene, tobacco, alcohol, nor concomitant diseases could be found to be associated with OSCC. Though, smoking was confirmed to be a factor associated with OSCC in univariate analysis with poor clinical relevance within the multivariate regression model.

## Conclusions

We could find hints for an association between radiological determined chronic periodontitis and OSCC. This finding is complemented by a possible beneficial effect of periodontal therapy that may possibly inhibit the occurrence of OSCC. Due to the high frequency of periodontal diseases worldwide, this aspect could be of high clinical relevance. Since the present data was gathered within a retrospective setting and a low response rate to the questionnaire, prospective clinical trials with focus on OSCC should take clinical periodontal parameter into account to furthermore support this potential intriguing aspect of cancer and inflammation.

## Endnote

^a^Data acquisition was part of the dissertation (Dr. med. dent.) of E. A.

## Competing interests

No funding has been available other than that of the author’s institution. The authors declare that they have no competing interests.

## Authors’ contributions

MM, EA, AK, VW and BA planned the study. MM, EA and AK carried out the patient selection and the measurement methods. VW conducted the statistical analysis together with PWK and BA. PWK, MM, AA and BA wrote the manuscript. All authors read and approved the final manuscript.
